# *pitx2* Deficiency Results in Abnormal Ocular and Craniofacial Development in Zebrafish

**DOI:** 10.1371/journal.pone.0030896

**Published:** 2012-01-27

**Authors:** Yi Liu, Elena V. Semina

**Affiliations:** 1 Department of Pediatrics and Children's Research Institute, Medical College of Wisconsin and Children's Hospital of Wisconsin, Milwaukee, Wisconsin, United States of America; 2 Department of Cell Biology, Neurobiology and Anatomy, Medical College of Wisconsin, Milwaukee, Wisconsin, United States of America; Instituto de Medicina Molecular, Portugal

## Abstract

Human *PITX2* mutations are associated with Axenfeld-Rieger syndrome, an autosomal-dominant developmental disorder that involves ocular anterior segment defects, dental hypoplasia, craniofacial dysmorphism and umbilical abnormalities. Characterization of the *PITX2* pathway and identification of the mechanisms underlying the anomalies associated with *PITX2* deficiency is important for better understanding of normal development and disease; studies of *pitx2* function in animal models can facilitate these analyses. A knockdown of *pitx2* in zebrafish was generated using a morpholino that targeted all known alternative transcripts of the *pitx2* gene; morphant embryos generated with the *pitx2^ex4/5^* splicing-blocking oligomer produced abnormal transcripts predicted to encode truncated pitx2 proteins lacking the third (recognition) helix of the DNA-binding homeodomain. The morphological phenotype of *pitx2^ex4/5^* morphants included small head and eyes, jaw abnormalities and pericardial edema; lethality was observed at ∼6–8-dpf. Cartilage staining revealed a reduction in size and an abnormal shape/position of the elements of the mandibular and hyoid pharyngeal arches; the ceratobranchial arches were also decreased in size. Histological and marker analyses of the misshapen eyes of the *pitx2^ex4/5^* morphants identified anterior segment dysgenesis and disordered hyaloid vasculature. In summary, we demonstrate that *pitx2* is essential for proper eye and craniofacial development in zebrafish and, therefore, that *PITX2/pitx2* function is conserved in vertebrates.

## Introduction

*PITX2* is a member of the *bicoid*-like homeodomain transcription factor family which, when mutated, is responsible for Axenfeld-Rieger syndrome (MIM ID #180500), an autosomal-dominant developmental disorder characterized by ocular anterior chamber anomalies, increased risk for glaucoma, dental hypoplasia, craniofacial dysmorphism and umbilical region abnormalities [1].

The majority of *PITX2* mutations are located in the homeobox region encoding the homeodomain and affect all known *PITX2* isoforms (see below). These mutations typically generate a null-allele, which supports *PITX2* haploinsufficiency as a mechanism for Axenfeld-Rieger syndrome, consistent with reports of gene deletion in some patients; *PITX2* mutations that appear to retain limited wild-type activities are usually associated with milder phenotypes [2]. Identification of increased transactivation activity of two mutant PITX2 proteins associated with human disease suggests that elevated PITX2 activity may also be disruptive [2], [3]. Expression studies demonstrate that *Pitx2* is expressed in neural-crest derived cells that contribute to the ocular and craniofacial tissues affected in Axenfeld-Rieger syndrome [1], as well as during brain, heart, lung, stomach, gut and gonad development [4]–[11].

Several *PITX2* isoforms have been reported with four transcripts, *PITX2A-D*, identified in humans [12], three, *Pitx2a–c*, in mice and frogs [13] and two, *pitx2a* and *pitx2c*, described in chickens [14], zebrafish [15] and even ascidians [16]; the isoforms have different N-terminal sequences but share exons encoding for the homeodomain and C-terminal region. Although some variations were observed, the *Pitx2* isoforms demonstrate largely overlapping expression patterns [13]–[15], [17]; similarly, functional assays demonstrate comparable DNA-binding and transactivation activities for the main isoforms with minor differences revealed under certain conditions [12], [18].

In the mouse, complete knockout of *Pitx2* results in embryonic lethality and ocular, craniofacial, dental, brain, heart, lung, body wall and other systemic defects; various conditional *Pitx2*-deficient animals were generated to study specific aspects of its function [7], [8], [10], [17], [19]–[21]. Ocular anomalies are observed in the *Pitx2−/−* homozygous but not in *Pitx2+/−* heterozygous animals and include arrest in anterior segment development, thickening of the mesothelial layer of the cornea resulting in enophthalmos, dysgenesis of the extraocular muscle and other defects. In terms of craniofacial anomalies, *Pitx2−/−* animals display arrested tooth development at the placode (for maxillary teeth) or bud (for mandibular teeth) stage. The combination of ocular and craniofacial defects observed in *Pitx2*-deficient mice shows similarity to Axenfeld-Rieger syndrome in humans, suggesting conservation of function for this gene during embryonic development.

Zebrafish offer several advantages compared to mammalian models including rapid early development, optical clarity, availability of gene manipulation techniques (including the recently developed gene knockout technnology via zinc-finger nucleases), and large number of progeny, therefore providing powerful genetic screening opportunities [22], [23]. Zebrafish have been utilized to demonstrate the effects of *pitx2* misexpression on heart development [5], [15] but the overall effects of *pitx2*-deficiency in zebrafish have not yet been fully investigated. In this manuscript, we demonstrate that the function of *pitx2* is conserved in vertebrates and that zebrafish *pitx2* is essential for proper eye and craniofacial development.

## Materials and Methods

### Animals

Zebrafish (*Danio rerio*) adult fish were maintained on a 14-hour light/ 10-hour dark cycle in system water; the embryos were acquired by natural spawning and kept at 28.5°C. The developmental stage was determined by time (hours post fertilization (hpf) or days post fertilization (dpf)) as well as by morphological criteria [24]. The *Tg (fli1:gfp)*, *Tg (gsnl1:gfp)* and *p53−/−* zebrafish lines used in this study have been previously described [25]–[27].

The study was carried out in accordance with the recommendations in the Guide for the Care and Use of Laboratory Animals of the National Institutes of Health. The protocol was approved by the Institutional Animal Care and Use Committee at the Medical College of Wisconsin (protocol number AUA00000352).

### In situ hybridization

Plasmids for *dlx2a*, *dlx4a*, *foxd3* and *lmx1b* were obtained from Open Biosystems (Thermo Fisher Scientific Inc, Huntsville, AL ); *pax6a* was kindly shared by Dr. Link (Medical College of Wisconsin, Milwaukee, WI); *pitx3* probe have been previously described [28]; *pitx2*-exon 5, *foxc1a* and *dkk2* plasmids were constructed by PCR amplification and cloned into pCRII-TOPO vector (Invitrogen, Carlsbad, CA) using with the following primers: *zf-pitx2ac-1345f*, 5′-CGAAGATTCGAACGATGACC-3′ and *zf-pitx2ac-1345r*, 5′-TGGGATGTTGAAAAACGAAA-3′ (to produce a 1345-bp probe that detects both *pitx2* isoforms); *zf-foxc1a-273F* and 5′-GAAACCCCCGTACAGCTACA-3′, *zf-foxc1a-273R*, 5′-AAAAAGCTGCCGTTCTCAAA-3′ (to produce a 273-bp probe); *zf_dkk2_ex1f503*, 5′- CACACTGGCAGAGGAGATCA and *zf-dkk2_ex3r503*: TCTGCTGTGGTTTTGACAGG (to produce a 503-bp probe). Digoxigenin-labeled RNA probes were synthesized by in vitro transcription and *in situ* hybridization was performed as previously described [28].

### Morpholino injections

Morpholino oligonucleotides were synthesized by Gene Tools (Gene Tools, Philomath, OR). Antisense morpholino oligonucleotides were designed to target translation initiation sites of the *pitx2a*, 5′-CAAGTTTGCGGCAGTGGGAGTCCAT-3′ (*pitx2a^ATG^*), and *pitx2c*, CCTTCATAGAGGTCATGGATAAGAC (*pitx2c^ATG^*), isoforms as well as the *pitx2* exon 4 donor site, 5′-TTTATCAAACTTACTCGGACTCTGG-3′ (*pitx2^ex4/5^*), and a standard control morpholino, 5′-CCTCTTACCTCAgTTACAATTTATA-3′ (*control-MO*), was used. The morpholinos were solubilized in water and diluted to 300–500 µM with 0.1% phenol red for microinjections into embryos. The morpholino oligomers (4–14 ng) were injected into 1–4 cell-stage embryos as previously described [28]. The efficacy of morpholino injections was evaluated by semi-quantitative RT-PCR using RNA extracted from injected embryos.

### Nucleic acid extractions and amplification

RNA was extracted using zebrafish embryos harvested in Trizol reagent (Invitrogen, Carlsbad, CA) and standard procedures. cDNA was synthesized from 1 µg of total RNA with SuperScript III (Invitrogen, Carlsbad, CA) and random hexamers. Semi-quantitative PCR was performed using cDNA prepared from whole embryos, uninjected wild-type, *pitx2^ex4/5^* and *control-MO* injected embryos, and using two sets of *pitx2* primers (a combination of *pitx2* primers 1 and 3 (499-bp product) and a combination of primers 2 and 3 (637-bp product to amplify isoforms *pitx2a* and *pitx2c*, respectively) and a control set of primers for *β-actin* (329-bp product). Oligonucleotide sequences are as follows: *pitx2* forward primer 1 (*zpitx2aF*): 5′-AAGACTGGCACGGCTGCAAA-3′, *pitx2* forward primer 2 (*zpitx2cF*): 5′-CGTCGGTAAATCGCTTGTCT-3′ and *pitx2* reverse primer 3 (*zpitx2-R*): 5′-AACTTGATCCTGGTACACCG-3′, and control forward and reverse primers: *β-actin-F*, 5′-GAGAAGATCTGGCATCACAC-3′, *β-actin-R*, 5′- ATCAGGTAGTCTGTCAGGTC-3′. The DNA sequence of PCR products was determined by direct automated DNA sequencing. DNA sequencing was performed to confirm the identity of PCR products by comparison with database records; no novel sequences are reported.

### Histological analysis, alcian blue and alkaline phosphatase staining

Histological analysis was performed using standard protocols. Briefly, embryos were fixed in 4% paraformaldehyde at 4°C, washed three times in PBS (pH 7.4), dehydrated in ascending grades of ethyl alcohol (25%, 50%, 75%, 100%) and infiltrated overnight in the dark at 4°C. The embryos were placed in a molding cup tray capped by an EBH-2 Block Holder (Electron Microscopy Sciences, West Chester, PA), embedded in a fresh mixture of embedding solution and polymerized at 4°C overnight according to the manufacturer's directions. Polymerized blocks were cut at 1–2 mm using a Leica RM2255 microtome (Leica Microsystems, Vienna, Austria) with glass knives made from 7.0 mm glass strips on a Leica EMKMR2 Knifemaker (LeicaMicrosystems, Vienna, Austria); sections were collected onto charged glass slides and dried on the heater at 150°C. Hematoxylin and eosin staining was performed using standard protocols. Alcian blue staining was performed according to Barrallo-Gimeno et al. [29]; cartilage elements were identified as previously described [30]. The alkaline phosphatase staining of hyaloid vessels was performed according to Alvarez et al. [31]. Images were obtained using an AxioImager Zeiss microscope (Zeiss, Thornwood, NY).

### Immunohistochemistry

Immunohistochemistry was performed with the following primary serum: mouse anti-Keratin Sulfate Proteoglycan antibody (Chemicon International, Temecula, CA), mouse anti-Keratan sulfate monoclonal antibody (Millipore, Billerica, MA), rabbit anti-crystallin αA and βB1 antibodies (a generous gift from Dr. Thomas C. Vihtelic); and the following secondary antibodies, Alexa Fluor 568 donkey anti-mouse IgG (H+L) (Invitrogen, Carlsbad, CA), Alexa Fluor 488 donkey anti-rabbit IgG (H+L) (Invitrogen, Carlsbad, CA) and FITC conjugated Affinipure donkey anti-mouse IgG (H+L) (Jackson Immuno Research Laboratories Inc, West Grove, PA). Embryos were fixed in 4% paraformaldehyde/PBS and infiltrated with 30% sucrose and then OCT, embedded in cryomolds and frozen. 7-µm sections were prepared, mounted on charged glass slides and dehydrated for 30 min at 37°C. Sections were incubated in blocking solution (1× PBS/10% normal donkey serum /0.1% Tween 20) for 1 hour at room temperature and then overnight at 4°C in primary antibody/blocking solution. The sections were washed in 1× PBS/0.1% Tween 20 and incubated for 1 hour at room temperature in blocking solution and then exposed to secondary antibody. Immunoreactivity was captured with a AxioImager Zeiss microscope (Zeiss, Thornwood, NY).

## Results

### Generation of a complete knockdown of *pitx2* in zebrafish by blocking translation or mRNA splicing

**Figure 1:**
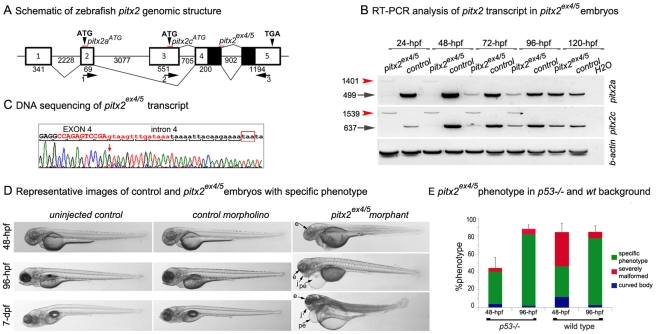
zebrafish *pitx2* knockdown and associated phenotype. **A**. Schematic drawing of *pitx2* genomic structure. Exons are shown as numbered boxes, sizes are indicated at the top (for exons) or at the bottom (for introns). The positions of primers to amplify *pitx2* transcripts are shown and numbered 1–3; primers 1 and 3 are used for *pitx2a* and 2 and 3 for *pitx2c*. The position of antisense morpholino oligonucleotides, *pitx2a^ATG^*, *pitx2c^ATG^* and *pitx2^ex4/5sp^*, are shown with red lines. **B**. RT-PCR of *pitx2* expression in *pitx2^ex4/5^* morphants. Please note a complete absence of normal *pitx2* transcripts (indicated with black arrows) and the presence of an abnormal large PCR product (indicated with red arrowheads) in mRNA extracted from *pitx2^ex4/5^* embryos at 24–48-hpf, the presence of both normal (diminished) and abnormal products at 72–96-hpf *pitx2^ex4/5^*, and normal levels of *pitx2* by 120-hpf due to weakening of morpholino effects. **C**. DNA sequencing of the abnormal PCR product observed in *pitx2^ex4/5^* morphant embryos identified the presence of the 902-bp intron 4 in the *pitx2^ex4/5^* transcript consistent with aberrant splicing (forward sequence is shown and the beginning of the intron is indicated with a red arrow; exon 4 sequence is shown in upper case while intron 4 is in lower case letters; the exon-intron junction sequence corresponding to the *pitx2^ex4/5^* antisense oligomer is indicated in red). Therefore, the *pitx2^ex4/5^* protein is predicted to contain partial *pitx2* sequence (lacking amino acids encoded by exon 5) followed by 10 erroneous amino acids (*pitx2^ex4/5^* stop codon is indicated with red box). **D**. Representative images of *pitx2^ex4/5^* morphants, control morpholino-injected embryos and larvae developed from uninjected eggs at 48-hpf, 96-hpf and 7-dpf. **E**. Bar graph showing the distribution of observed embryonic phenotypes following *pitx2^ex4/5^* morpholino injections into *p53*−/− and wild-type zebrafish eggs. e- eye, j- jaw, pe- pericardial edema.

The zebrafish *pitx2* gene generates two transcripts, *pitx2a* and *pitx2c*, produced by alternative promoter usage similar to the human *PITX2* gene [1], [15] (Figure 1A). Both *pitx2* transcripts are expressed embryonically at various sites including the developing pharyngeal arches, tissues surrounding the oral cavity and anterior segment of the eye [15], [32].

To achieve a complete knockdown of *pitx2*, the following antisense morpholino oligomers were designed: *pitx2a^ATG^* and *pitx2c^ATG^* (targeting the translation initiation sites of the *pitx2a* and *pitx2c* isoforms, respectively) and *pitx2^ex4/5^* (targeting the donor site of exon 4 and predicted to affect normal splicing of exons 4 and 5 that are common to both *pitx2a* and *pitx2c* transcripts and, therefore, to simultaneously knockdown both *pitx2* isoforms) (Figure 1A). Injection of either a combination of *pitx2a^ATG^/pitx2c^ATG^* oligomers or the single *pitx2^ex4/5^* morpholino resulted in an abnormal phenotype, described below. RT-PCR analysis of mRNA extracted from *pitx2^ex4/5^* morphants identified an acute reduction (in embryos injected with 4.7 ng of *pitx2^ex4/5^* morpholino) or a complete absence (in larvae injected with 7 ng of morpholino) of normal *pitx2* transcripts along with the presence of an aberrant PCR product at 24–48-hpf (Figure 1B); the quantity of normal transcript continued to be significantly diminished in 72–96-hpf *pitx2^ex4/5^* morphants and reached normal levels in 120-hpf embryos (Figure 1B). Sequence analysis of the aberrant RT-PCR product revealed the presence of a 902-bp fragment corresponding to intron 4 (Figure 1C). The abnormal transcript was predicted to encode truncated pitx2a and pitx2c proteins lacking the third (recognition) helix of the DNA-binding homeodomain with pitx2a sequence truncated at 92 amino acids (34% of normal length) and pitx2c at 137 amino acids (44% of normal length).

### Knockdown of *pitx2* disrupts embryonic development

Embryos injected with either a combination of *pitx2a^ATG^* and *pitx2c^ATG^* oligomers or a single *pitx2^ex4/5^* morpholino demonstrated similar abnormal phenotypes, indicating an essential role for *pitx2* in normal zebrafish development; *pitx2^ex4/5^* morpholino was selected for further studies as it provided the ability to easily verify/quantify injection outcomes by RT-PCR (please see above; Figure 1B) and injection of this single oligomer was associated with higher efficiency and lower mortality rates.

Gross morphological comparison of *pitx2^ex4/5^* morphant and control embryos was performed using 150–300 embryos in each experiment: at 24-hpf, the first embryos displaying smaller head, eyes and body length were detected with the number of abnormal embryos increasing sharply by 48-hpf; most of these embryos survived until 6–8-dpf and, in addition to the small eye, head, and trunk, displayed pericardial edema (starting at ∼60-hpf) and jaw abnormalities (from ∼72-hpf) (Figure 1D). Other phenotypic classes included severely malformed fish (displaying twisted shortened trunk, small malformed head, massive heart edema, and associated with lethality at ∼72-hpf in most fish), “curved body” fish, and embryos with normal appearance. The proportions of phenotypes were as follows: specific head and trunk and severe phenotypes were observed in 70–82% of *pitx2^ex4/5^* morphants and not observed in embryos injected with control morpholino or uninjected larvae; the non-specific “curved body” phenotype was detected in ∼12% of *pitx2^ex4/5^*, 14–16% of control morpholino-injected larvae, and not observed in uninjected embryos; normal phenotype was seen in 6–16% of *pitx2^ex4/5^* morphants, 84–86% of control morpholino-injected fish and ∼100% of uninjected embryos. Since the “curved body” fish were present at a similar rate in *pitx2^ex4/5^* morphants and control embryos, this phenotype was classified as non-specific and further studies focused on morphants with specific head/trunk or severely malformed phenotypes.

To further investigate the specificity of the observed *pitx2^ex4/5^* phenotypes, injections into *p53*-deficient zebrafish embryos were performed to identify features possibly associated with off-target effects which may accompany morpholino injections due to activation of p53-mediated apoptosis [27]. The distribution of observed phenotypes following *pitx2^ex4/5^* injections was similar at the 96-hpf stage while at 48-hpf a lower percentage of “curved body” (4% versus 12%) and severe phenotypes (4.3% versus 38.4%) was detected in *p53−/−* versus wild-type backgrounds (Figure 1E). These data suggest that both the “curved body” and severe phenotypes detected at earlier stages may be related to off-target effects of morpholino injections while the specific head and trunk phenotype observed at both earlier and later stages is caused by *pitx2* deficiency. Further analyses of morphology, histology and alcian blue staining were performed using zebrafish that displayed the specific phenotype in wild-type and *p53−/−* backgrounds with no apparent difference in features observed in these two backgrounds.

### *pitx2 ^ex4/5sp^* morphants demonstrate craniofacial and ocular defects

To investigate the craniofacial and ocular anomalies associated with *pitx2* deficiency, *pitx2^ex4/5^* morphant and control embryos were assayed by alcian blue staining and histological analysis.

**Figure 2:**
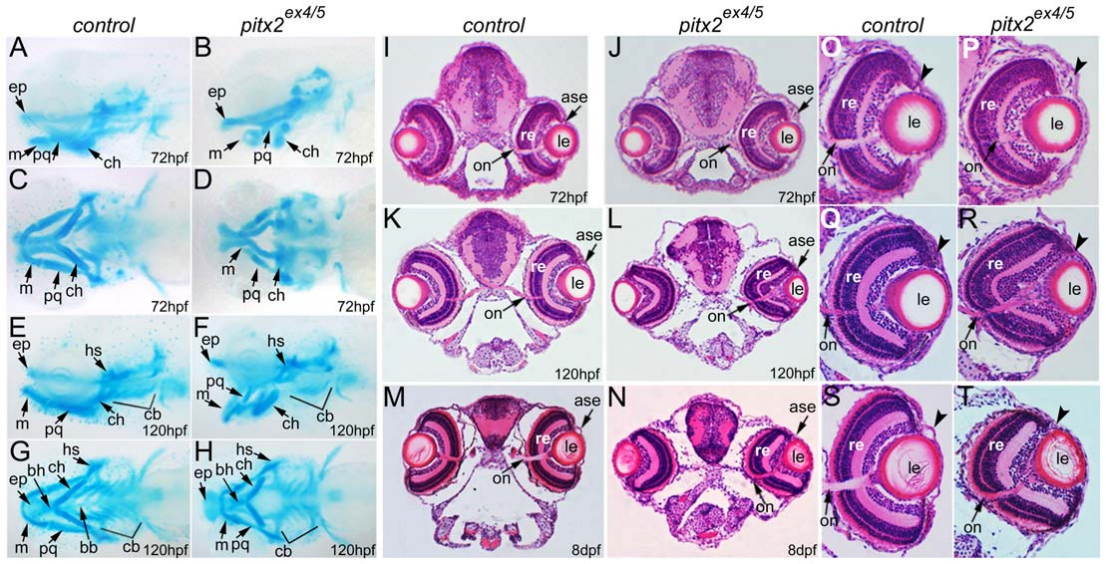
Deficiency of *pitx2* results in craniofacial and ocular defects. **A–H**. *pitx2* knockdown disrupts development of the pharyngeal arches. Lateral (**A, B, E, F**) and ventral (**C, D, G, H**) views of 72-hpf (**A–D**) and 120-hpf (**E–H**) larvae stained with Alcian blue. Numerous defects in the development of the lower jaw (anterior arches) and ceratobranchial (posterior) arches can be observed. **I–T**. *pitx2* knockdown disrupts eye development. Representative images of histological sections of 72-hpf (**I,J,O,P**), 120-hpf (**K,L,Q,R**) and 8-dpf (**M,N,S,T**) control and *pitx2^ex4/5^* morphants; head sections at the optic nerve level are shown (**I–N**) as well as high magnification images for the eye (**O–T**). Please note the accumulation of cells in the anterior segment in 72-hpf *pitx2^ex4/5^* morphant embryos (arrowhead in **P**) and abnormal eye size/shape in 120-hpf and 8-dpf morphants (arrowheads in **R**, **T**). Abbreviations: **A–H**- bb,basibranchial; bh, basihyal; cb, ceratobranchial (P3–P7); ch, ceratohyal (P2); ep, ethmoid plate; hs, hyosympletic (P2); m, Meckel's cartilage (P1); pq, palatoquadrate (P1); **I–T**- ase, anterior segment of the eye; le, lens; on, optic nerve; re- retina.

The cartilage elements of the *pitx2^ex4/5^* pharyngeal arches were reduced in size, malformed and displaced (Figure 2A–H). This phenotype was clearly visible starting at 72-hpf; no noticeable abnormalities were detected in morphant embryos at earlier stages of development using morphological and histological analyses. The position of the palatoquadrate of the mandibular arch (or the first pharyngeal arch) appeared to be normal while the Meckel's cartilage component of the mandibular arch was positioned perpendicular to the palatoquadrate and pointing towards the upper jaw in 72-hpf embryos (Figure 2A–D). The ceratohyal cartilage of the hyoid arch (or the second pharyngeal arch) was positioned perpendicular to the upper jaw and pointing ventrally. The ceratobranchial arches 1 through 5 (pharyngeal arches 3–7) as well as the basihyal and basibranchial components of the pharyngeal arches appeared to be underdeveloped. At 120-hpf (Figure 2E–H), the mandibular and the hyoid pharyngeal arches were pointed ventrally, giving the appearance of an “open mouth”. In addition to this, a visible reduction in size and irregular shape of the elements of the mandibular arch (Meckel's and palatoquadrate cartilages) was noted. The ceratobranchial arches could be observed at this stage but were reduced in size. At the same time, the ethmoid plate cartilage of the upper jaw appeared to be largely unaffected. The described anomalies were observed in 100% of 72–120-hpf *pitx2^ex4/5^* morphants (n = 35 (72–96-hpf), n = 20 (120-hpf) embryos).

Analysis of histological sections at the eye level revealed defects in ocular development in *pitx2^ex4/5^* morphants (Figure 2I–T). At 72-hpf, the developing eye appeared to be largely unaffected with the exception of an increased number of cells observed in the anterior segment in some embryos (40%, n = 30; Figure 2I, J, O, P). At 120-hpf and 8-dpf, the *pitx2^ex4/5^* eyes seemed small/misshapen and appeared to lack developed anterior segment region structures (93%, n = 30 (120-hpf) and 100%, n = 10 (8-dpf); Figure 2K–N and Q–T).

**Figure 3:**
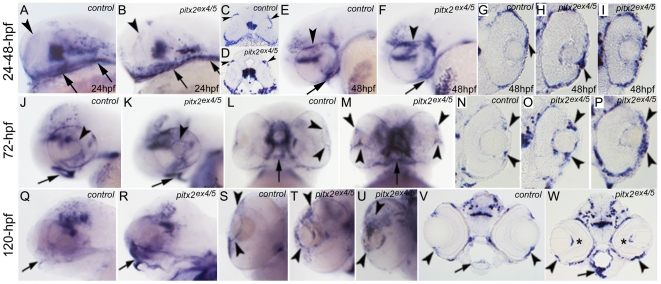
In situ hybridization with *pitx2-exon 5* antisense riboprobe in control and morphant embryos. The *pitx2* antisense riboprobe comprising exon 5 sequence detects wild-type and abnormally spliced *pitx2^ex4/5^* transcripts. Images of whole mount embryos (**A–F**, **J–M** and **Q–U**) and sections (**G–I**, **N–P** and **V, W**) are shown for embryonic stages of 24-hpf (**A–D**), 48-hpf (**E–I**), 72-hpf (**J–P**) and 120-hpf (**Q–W**). Please note abnormal *pitx2* transcripts around the developing eye (arrowheads in **B, D**) and pharyngeal arches (arrows in **B**) in morphants at 24-hpf, which is similar to *pitx2* expression in control embryos (**A**, **C**). Staining for *pitx2* positive cells in morphant embryos at 48-, 72- and 120-hpf identifies abnormal patterns during ocular and craniofacial development in comparison to *pitx2* expression in control embryos (**E–W**). In terms of ocular patterns, some 48- and 72-hpf morphants demonstrate an accumulation of *pitx2* transcriptionally active cells in the anterior segment of the eye (arrowheads; images for whole mount and sections from two different *pitx2^ex4/5^* morphant embryos at 48-hpf (**F, H, I**) and 72-hpf (**K, M, O, P**) in comparison to control 48-hpf (**E,G**) and 72-hpf (**J, L, N**) are shown). In 120-hpf eyes, a disorganized pattern of *pitx2* positive cells continues to be observed in *pitx2^ex4/5^* morphant embryos (arrowheads in **T**, **U**, two different morphant embryos are shown, and **W**) in comparison to *pitx2* expression in control embryos (**S, V**); in addition to the abnormal pattern in the anterior structures, an increased signal behind the lens corresponding to the hyaloid vasculature is also observed (asterisks in **W**). With regards to craniofacial development, in 72-hpf morphant embryos, strong staining is observed around the malformed oral cavity and arches with the level of expression similar to control *pitx2* expression (arrows in **J–M**); at 120-hpf, *pitx2* transcripts continue to be strongly expressed in the malformed pharyngeal arches (arrows in **R** and **W**) while expression of wild-type *pitx2* is downregulated in control 120-hpf embryos (arrows in **Q** and **V**).

Since *pitx2^ex4/5^* morphant embryos express abnormally spliced *pitx2* transcript that includes exon 5, we were able to use the *pitx2*-exon 5 antisense riboprobe to detect cells with active *pitx2* expression in morphant and control embryos and to compare these patterns: in early stage (24–48-hpf) *pitx2^ex4/5^* embryos, we expected to primarily observe abnormal transcript while at later stages (72–120-hpf) we were likely to detect a mixture of abnormal and normal *pitx2* transcripts due to weakening of the effects of morpholino in the maturing *pitx2^ex4/5^* embryos. At 24-hpf, *pitx2-*positive cells were detected in morphants in a pattern similar to normal *pitx2* expression in control embryos in the brain, developing pharyngeal arches and around the eye (Figure 3A–D). At 48-hpf, the overall pattern continued to be similar but increased *pitx2* staining was observed in the anterior segment of the eye on sections (80% of morphants, n = 10; Figure 3E–I) and by 72-hpf, an abnormal accumulation/pattern of *pitx2* positive cells was detected by both whole mount and section in situ hybridization (100% of morphants, n = 17; Figure 3J–P). Clusters of *pitx2* positive cells in the anterior segment of the eye were seen as late as 120-hpf (100% of *pitx2 ^ex4/5^* embryos, n = 10; Figure 3Q–W); an increased staining behind the lens, in the developing hyaloid vasculature was also observed (Figure 3W). Similarly, robust expression of *pitx2* in the craniofacial region of morphant embryos was observed at 72-hpf and 120-hpf in contrast to downregulation of normal *pitx2* expression in control embryos (100% of morphants, n = 17 (72-hpf), n = 10 (120-hpf); Figure 3J–W).

### Patterns of craniofacial and ocular gene expression are affected in *pitx2^ex4/5^* morphants

In vertebrate embryos, the pharyngeal arches receive a significant contribution from the cranial neural crest cells that migrate into the craniofacial region. Forkhead transcription factor *foxd3* is required for maintenance of neural crest population [33], [34], *fli1* is expressed in neural crest derived lineages [33] and the distal-less homeobox gene family plays an important role in early as well as late stages of craniofacial development [35], [36]. We analyzed expression of *foxd3*, *dlx2a* and *dlx4a*, and *Tg(fli1:gfp)* in *pitx2^ex4/5^* morphants and control embryos and observed a number of differences.

**Figure 4:**
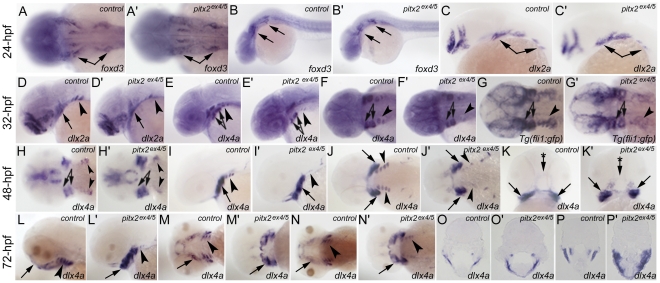
Craniofacial development and gene expression in *pitx2^ex4/5^* morphants. Developmental patterns of *foxd3*, *dlx2a* and *dlx4a*, and *Tg(fli1:gfp)* expression in *pitx2 ^ex4/5^* embryos. In situ hybridization was performed with *foxd3*, *dlx2a*, *dlx4a* or *gfp*-specific antisense riboprobe in control (**A–P**) or morphant (**A′–P′**) zebrafish embryos. Please note similar expression patterns in migrating neural crest cells (*foxd3*) and pharyngeal arch primordial regions (*dlx2a*) at 24-hpf (arrows in control (**A–C**) and morphant (**A′–C′**) embryos). Starting at 32-hpf, expression of *dlx2a*, *dlx4a* and *Tg(fli1:gfp)* in the posterior arches appears to be reduced in *pitx2^ex4/5^* fish (arrowheads in control (**D–G**) and morphant (**D′–G′**) embryos) while expression in the anterior arches (arrows in control (**D–G**) and morphant (**D′–G′**) embryos) is not significantly affected. In 48–72-hpf embryos, an increased expression of *dlx4a* in the anterior arches is detected (arrows in control (**H–N**) and morphant (**H′–N′**) embryos) while expression in the posterior arches remains reduced (arrowheads in control (**H–N**) and morphant (**H′–N′**) embryos); *dlx4a* expression around the oral cavity in 48-hpf embryos (arrow with asterisk in **K** and **K′**) as well as a frontward extension of the anterior arches at 72-hpf (arrows in **L–N** for controls and **L′–N′** for morphants) are not observed in *pitx2^ex4/5^* fish. Analysis of sections at 72-hpf also shows broadened expression of *dlx4a* in the craniofacial region of morphant embryos (**O′, P′**) in comparison to controls (**O, P**).

Expression of *foxd3* in 24-hpf *pitx2^ex4/5^* embryos appeared to be mostly unaffected with similar levels of transcripts seen in the migrating neural crest cells marked with *foxd3* (Figure 4A/A′ and B/B′); expression of *dlx2a* was similar in regions of the anterior (mandibular and hyoid) arches while slightly reduced levels were seen in the posterior (ceratobranchial) arches (n = 10; Figure 4C and C′). At 32-hpf, a decrease in signal associated with the posterior arches was clearly observed in ∼80% of morphant embryos based on expression of *dlx2a*, *dlx4a* and *Tg(fli1:gfp)* (n = 5–8; Figure 4D–G and D′–G′). By 48-hpf, an increased *dlx2a* staining in the dorsal domain of the anterior arches and reduction of staining in the posterior arches was observed in 92.5% of morphant embryos (n = 25; Figure 4H and H′). Expression of *dlx4a* in the developing pharyngeal arches and in the epithelia around the oral cavity at 48-hpf was also affected: expression in the posterior arches or anterior domain of the oral cavity was decreased while broader expression was detected in the anterior arches (65% of morphants, n = 17; Figure 4I–K and I′–K′). In 72-hpf control embryos, *dlx4a* expression was detected in the anterior arches that extend frontward as well as in the posterior arches, while lack of the anterior extension is observed in *pitx2^ex4/5^* morphants with broad strong *dlx4a* expression continuing in this region in 83% of morphants (n = 23; Figure 4L–P and L′–P′).

Expression of several ocular factors was also examined in *pitx2^ex4/5^* morphants. This analysis included *pax6a*, a paired-box homeodomain transcription factor [37]; *pitx3*, another member of the *pitx* family that plays an active role in early lens development [28], [38]; *dkk2*, a zebrafish ortholog of mammalian *Dkk2* that encodes an extracellular antagonist of canonical Wnt/ß-catenin signaling and is downstream of Pitx2 in the developing eye in mice [39]; corneal keratan sulfate proteoglycan that is highly expressed in the corneal stroma and represents an excellent cornea-specific differentiation marker [40], crystallins αA and βB1 that are expressed in the developing lens fibers [41], [42] and *Tg (gsnl1:gfp)* that marks the developing annular ligament (analog of trabecular meshwork) in the iridocorneal angle of zebrafish eyes [26].

**Figure 5:**
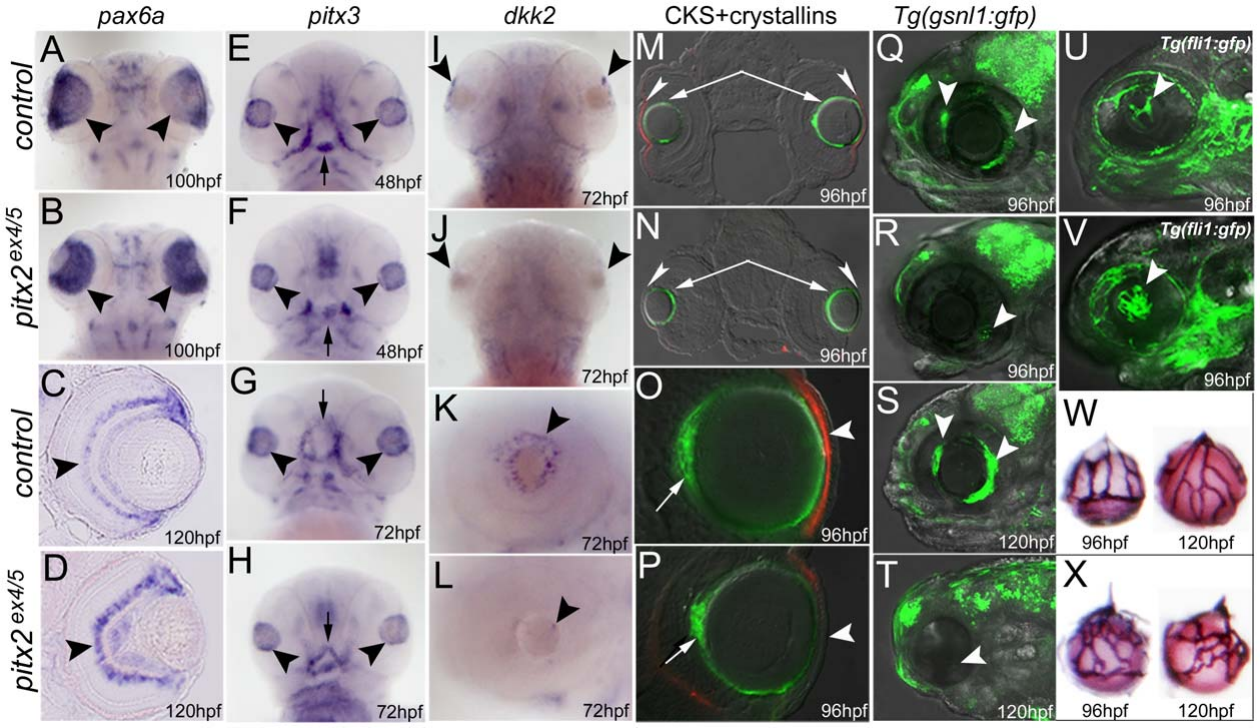
Ocular development and marker expression in *pitx2^ex4/5^* morphants. In situ hybridization (**A–L**), double immunohistochemistry (**M–P**), live gfp fluorescence images (**Q–V**) and alkaline phosphatase staining (**W, X**) for control and morphant embryos are shown and are labeled with corresponding developmental stages in the lower right corner; dorsal (**A**, **B**, **I**, **J**) and ventral (**E**–**H**) views of a zebrafish larval head as well as transverse sections at the eye level (**C**, **D**, **M**–**P**), all positioned anterior to the top, and lateral views of an embryonic eye (**K**, **L**) or head (**Q**–**V**), positioned anterior to the left, are shown. Please note expression of *pax6a* in the developing retina of the control and morphant embryos (arrowheads in **A–D**); similar *pitx3* expression in the developing lens in 48–72-hpf control and morphant embryos (arrowheads in **E–H**; abnormally developing pharyngeal arches and oral cavity in 48–72-hpf embryos are marked with arrows); reduced expression of *dkk2* in the anterior segment of the *pitx2* morphants (arrowheads in **I–L**); decreased/absent expression of corneal keratan sulfate proteoglycan (CKS) in the developing morphant corneas (red fluorescence, arrowheads in **M–P**) but similar expression of crystallins αA (green fluorescence, arrows in **M, N**) and βB1 (green fluorescence, arrows in **O, P**) in control and morphant lenses; and a decreased/absent expression of the *Tg(gsnl1:gfp)* transgene in the developing iridocorneal structures of *pitx2^ex4/5^* morphants (arrowheads in **R, T**) in comparison to control embryos (**Q, S**). In addition to this, fluorescence (*Tg(fli1:gfp*) transgene) images (**U, V**) as well as alkaline phosphatase staining (**W, X**) demonstrate abnormal development of the hyaloid vasculature in *pitx2^ex4/5^* morphants (arrowhead in **V**) in comparison to control embryos (**U**); increased number and disorganized appearance of *pitx2^ex4/5^* hyaloid vessels (**X**) in comparison to controls (**W**) is revealed by alkaline phosphatase staining.

Expression of *pax6a* was detected in the retinal ganglion cells and inner nuclear layer neurons in *pitx2^ex4/5^* morphant and control 100–120-hpf eyes (Figure 5A–D). Expression of *pitx3* in the developing lens also seemed to be mostly unaffected, although morphant lenses appeared to be smaller than control lenses (n = 6; Figure 5E–H); in addition to the lens, *pitx3* is expressed in the developing pharyngeal arches in a pattern similar to *pitx2* and this expression continued to be observed in the abnormally developing craniofacial structures (Figure 5F and H).

The most significant changes were detected in the expression of genes associated with the developing anterior segment structures. Analysis of *dkk2* expression in *pitx2^ex4/5^* morphants revealed a sharp reduction in the amount of *dkk2* transcript in the anterior segment region at 72-hpf (67% of *pitx2^ex4/5^* embryos; n = 12) (Figure 5I–L). Dual immunohistochemistry of keratan sulfate proteoglycan and crystallin (αA and βB1) in control and *pitx2^ex4/5^* embryos at 96-hpf demonstrated normal expression for both crystallins in 100% of the *pitx2^ex4/5^* lenses (n = 16) while highly reduced/absent staining for keratan sulfate proteoglycan was observed in 75% of *pitx2^ex4/5^* corneal stroma (n = 16; Figure 5M–P). Examination of GFP expression in Tg (*gsnl1*:gfp) zebrafish embryos injected with control or *pitx2^ex4/5^* morpholino identified clearly detectable patches of GFP positive cells in the iridocorneal angle and the developing annular ligament in 72–96-hpf eyes followed by strong expression in all 120-hpf control embryos (n = 10 for every stage) while a significant reduction (80%) or a complete absence (20%) of ocular GFP expression was observed in *pitx2^ex4/5^* 72–120-hpf morphants (n = 10) (Figure 5Q–T).

Finally, the development of the ocular vasculature in *pitx2^ex4/5^* morphants was evaluated using the Tg*(fli1:gfp)* transgenic line [25]; in this line, the *fli1* promoter drives the expression of green fluorescent protein (gfp) in all blood vessels throughout embryogenesis. In 100% of 72–120-hpf *pitx2^ex4/5^* morphants, the ocular blood vessels appeared to be disorganized and misdirected based on fluorescence detection (n = 15; Figure 5U, V) and in situ analysis with the *gfp* probe (data not shown). To further investigate the hyaloid vasculature patterning in control and *pitx2^ex4/5^* morphants, alkaline phosphatase staining was performed as described [31]. Consistent with the *Tg(fli1;gfp)* data, *pitx2^ex4/5^* hyaloid vessels demonstrated abnormal branching with extra interconnections, appeared to be thinner, were increased in number and attached loosely to the smaller lens in comparison to control lenses (Figure 5W, X). The size of the *pitx2^ex4/5^* lens was measured and found to be significantly smaller than the control lens (p = 0.034 for 72-hpf and p = 0.007 for 120-hpf).

## Discussion

In this manuscript we describe the effects of *pitx2* knockdown in zebrafish. The functional disruption generated with *pitx2^ex4/5^* splice-blocking morpholino is highly similar to the effects of human *PITX2* mutations, which are clustered in the homeodomain region of the protein with many of them resulting in truncation of normal PITX2 sequence and lack of the recognition helix of the homeodomain [1]–[3]. Deficiency of *pitx2* led to a number of abnormalities in zebrafish including craniofacial and ocular defects consistent with the classic features of Axenfeld-Rieger syndrome. These data, together with the previously reported preservation of *PITX2/pitx2* expression pattern and transcriptional regulation [32] suggests a high level of conservation of *PITX2/pitx2* function in vertebrates.

The craniofacial abnormalities observed in *pitx2^ex4/5^* morphants appear to be similar to what was reported in *foxd3* and *dlx* mutants and morphants [34], [36]. We observed no significant differences in the expression pattern of *foxd3* in *pitx2^ex4/5^* morphants; in general, expression of *foxd3* appears to precede *pitx2* expression in the pharyngeal arches' primordial region and, therefore, foxd3 may be acting upstream of *pitx2* in the developmental pathway. PITX2 and *DLX2* have been previously reported to be acting in the same pathway during tooth development in mammals; PITX2 was shown to bind to *bicoid*-like sites in the *DLX2* promoter resulting in a 30-fold activation of reporter expression in transfected cells [43].

Ocular developmental phenotypes observed in *pitx2^ex4/5^* zebrafish such as accumulation of *pitx2*-transcriptionally active cells in the front of the developing eye followed by lack of differentiation of anterior segment structures are highly similar to the features previously reported in *Pitx2* knockout mice [8], [19] and point to possible defects in migration patterns and differentiation of neural-crest derived periocular mesenchymal cells. The expression pattern of several ocular markers was found to be affected in *pitx2^ex4/5^* morphants consistent with the abnormal development of anterior segment structures and overall ocular dysgenesis. In addition to anterior segment dysgenesis and vasculature defects in *pitx2* deficient zebrafish, a misshapen and small retina and lens were observed. A small and misshapen eye/retina has been previously reported in *Pitx2−/−* deficient mice [19]. At this point it is unclear whether pitx2 plays a direct role in the development of posterior ocular structures or if maldevelopment of the anterior segment and/or hyaloid vasculature affects the formation of other parts of the eye including the retina; the second possibility seems to be more likely since *pitx2* is not expressed in the developing retina.

Zebrafish *pitx2* deficiency was also found to result in an abnormal patterning of the hyaloid vasculature with an increased number of thin and disorganized vessels observed in the *pitx2 ^ex4/5^* morphant eyes. Recently, Rutland et al demonstrated that defects in the development of the hyaloid vasculature can lead to microphthalmia and lens anomalies in mice [44]. A similar mechanism may be implicated in the small eye/lens phenotype observed in *pitx2* deficient zebrafish reported here; the role of the hyaloid vasculature in normal development of various ocular structures needs to be investigated further. Previously, Evans and Gage [21] observed hypomorphic hyaloid vasculature in neural-crest-specific *Pitx2* knockout mice: a reduced number of hyaloid vessels was observed in the developing mutant eye that was apparently associated with an inability of the *Pitx2*-negative pericytes to differentiate and stabilize the developing vessels. Abnormalities in human ocular vasculature development such as persistent fetal vasculature are often associated with anterior segment defects, cataracts, glaucoma, retinal detachment, nystagmus, microphthalmia, and other ocular anomalies [45]–[47]. Recently, a persistent hyaloid artery extending towards the fibrovascular tissue behind the lens was identified in a patient affected with Axenfeld-Rieger syndrome caused by a *PITX2* mutation [48], further suggesting that human *PITX2* may be involved in normal ocular vasculogenesis, similar to its vertebrate orthologs.

The hyaloid vasculature represents a transient intraocular system which is gradually lost during the late embryonic period with a complete regression in adults; the embryonic eye relies on the hyaloid vasculature to provide nourishment to its various developing structures [31], [49], [50]. The mechanisms of hyaloid vasculogenesis and its subsequent regression are not yet well understood. Disorganized/ persistent hyaloid vasculature defects have also been previously reported in *Bmp4* [51], *plexin D1* [31], *Cited2* [52], *Lama1/lama1* [53], [54], *norrie disease protein (Ndp)* [55], *Wnt7b* [56] and *Frizzled-5* [57], [58] zebrafish or mouse mutants as well as in association with mutations in the human *LAMB2* [59], *NDP* [60] and *FRIZZLED-4* [61] genes. Interestingly, Cited2 has been previously reported to directly control *Pitx2c* expression during cardiovascular development [62]. *Pitx2c* expression in the left lateral plate mesoderm was absent in the *Cited2(−/−)* mice; Cited2 and Tfap2 were found to be bound to the *Pitx2c* promoter in embryonic heart tissues and to activate *Pitx2c* transcription in transient transfection assays. Therefore, it is possible that a similar pathway is acting during ocular vasculature development.

The Axenfeld-Rieger malformation spectrum represents a complex group of conditions showing significant intrafamilial and interfamilial variability [2], [3]. Ocular abnormalities appear to be the most penetrant feature of *PITX2*-deficient phenotypes as even partial loss-of-function mutations can result in ocular manifestations [2], [3]. The mechanisms of these ocular defects and, in particular, the developmental glaucoma seen in ∼50% of affected patients, are still unclear. Our manuscript introduces a zebrafish model of *pitx2* deficiency and presents data that support conservation of *pitx2* function in vertebrates. While our results are consistent with *pitx2* playing an essential role in anterior segment development, other ocular anomalies observed in zebrafish suggest a broader role for *pitx2/PITX2* in the developing eye, with potential implications to human disease, that needs to be investigated further. At the same time, the phenotype reported here is associated with a complete loss of normal *pitx2* at early developmental stages while the human phenotype, Axenfeld-Rieger syndrome, is caused by *PITX2* haploinsufficiency; it is still unclear whether permanent inactivation of one allele of *pitx2* can lead to a zebrafish phenotype with relevance to human disease. Since morpholino technology offers a temporary and imprecise disruption of gene function, the development/identification of a permanent zebrafish line carrying a *pitx2*-inactivating mutation is needed to fully investigate this question. Zebrafish are increasingly being utilized for studies of disease mechanisms due to a number of advantages in comparison to mammalian systems [22], [23]; studies of *pitx2-*deficient zebrafish are likely to facilitate examination of the developmental roles of this important factor.
